# Draft Genome Sequence of Agrobacterium radiobacter Strain MD22b, Isolated from a Grape Plant in Tajikistan

**DOI:** 10.1128/mra.01131-22

**Published:** 2023-03-20

**Authors:** Munavvara Dzhuraeva, Khursheda Bobodzhanova, Rubén Javier-Lopez, Marina Tediashvili, Ekaterine Jaiani, Nils-Kåre Birkeland

**Affiliations:** a Center of Biotechnology of the Tajik National University, Dushanbe, Tajikistan; b G. Eliava Institute of Bacteriophages, Microbiology and Virology, Tbilisi, Georgia; c Department of Biological Sciences, University of Bergen, Bergen, Norway; d School of Medicine, New Vision University, Tbilisi, Georgia; DOE Joint Genome Institute

## Abstract

Agrobacterium radiobacter strain MD22b was isolated from infected fruit from Vatan Farm, a dekhkan farm in Yangibog (Tursunzade, Tajikistan). The 5.7-Mbp draft genome sequence presented here shares homology with chromosomes 1 and 2, as well as with the Ti plasmid from agrobacteria.

## ANNOUNCEMENT

**FIG 1:**
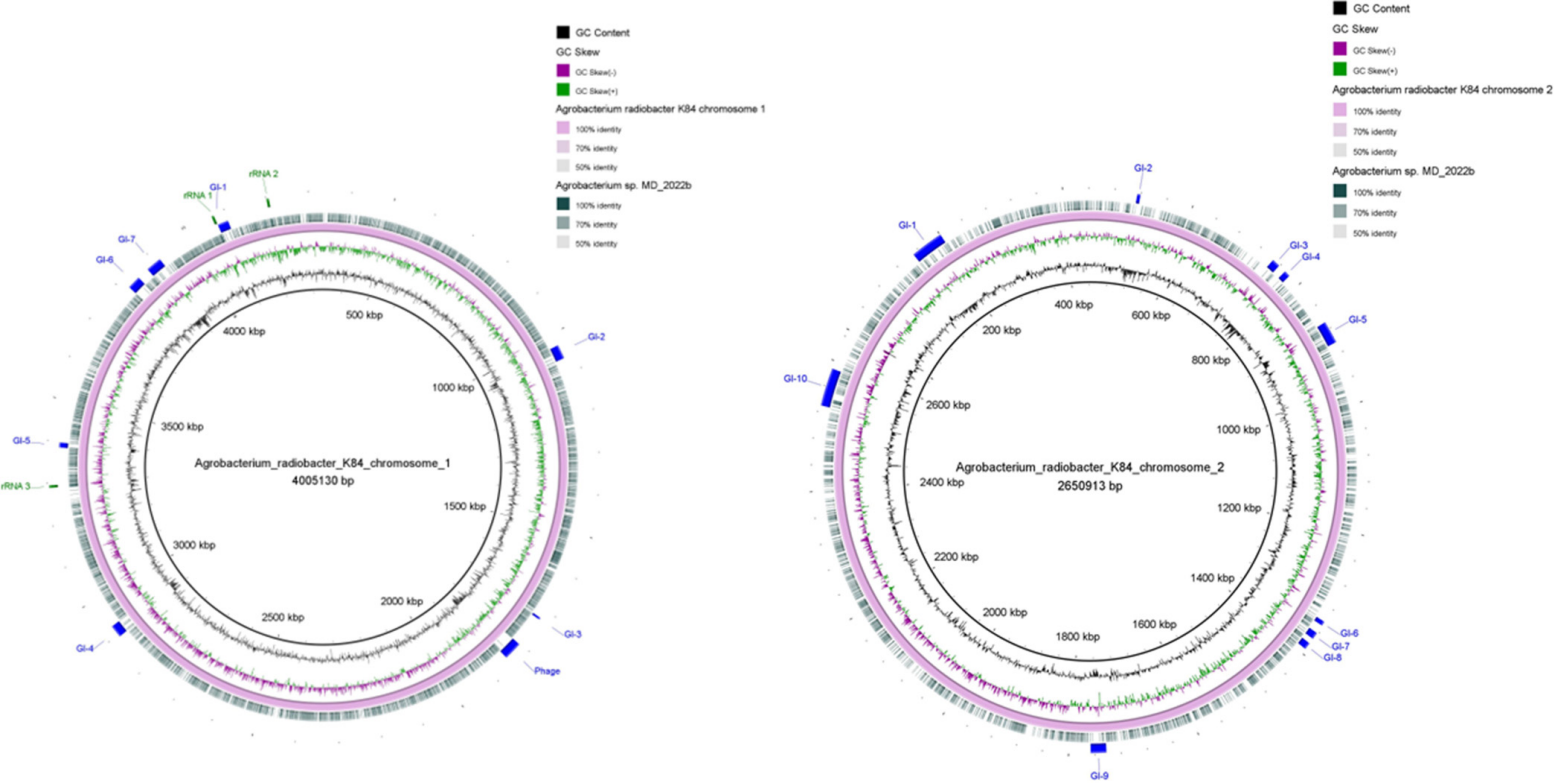
Circular representation of the MD22b genome compared with chromosomes 1 (left) and 2 (right) of the reference strain *A. radiobacter* K84, using the BLAST Ring Image Generator (BRIG) (10). The locations of genomic islands (GIs), prophage elements (Phage), a CRISPR/Cas element, and the rRNA genes in strain K84 are indicated.

Agrobacterium radiobacter (Beijerinck and van Delden 1902) Conn 1942 (formerly Agrobacterium tumefaciens) (1) was first isolated from grapevine galls in 1897 as the causative agent of crown gall disease (2). Despite their great importance in agriculture, strains of *Agrobacterium* from Tajikistan have not been studied previously. Within the framework of a project aiming at development of phage therapy for treatment of *Agrobacterium*-infected grapevines in Tajikistan, strain MD22b was isolated from an infected fruit collected in September 2019 at Vatan Farm, a dekhkan farm in Yangibog (Tursunzade, Tajikistan). The fruit was rinsed three times in sterile distilled water and homogenized. Aliquots were spread onto Roy-Sasser agar (3) plates and incubated at 30°C for 3 days. Colonies were picked and streaked onto fresh Roy-Sasser agar plates for purification. One dark-red *Agrobacterium*-like colony was picked and identified as *A. radiobacter* by its API 20 NE (bioMérieux) profile (1-4-6-7-7-4-4). DNA was extracted from cells cultivated in LB for 24 h at 30°C with shaking using the GenElute bacterial genomic DNA kit (Sigma-Aldrich). Sequencing of the 16S rRNA gene using the primer combination 5′-AGRGTTTGATYHTGGCTCAG-3′ (27f_mod) and 5′-TASGGHTACCTTGTTACGACTT-3′ (1492r_mod) (4) as previously described (5) yielded a 1,275-nucleotide sequence identical to that of *A. radiobacter* strain ATCC 23308 (GenBank accession number MT534521.1) using BLASTn v. 2.13.0 searches against the GenBank v. 252 nonredundant nucleotide database (https://blast.ncbi.nlm.nih.gov/Blast.cgi?PROGRAM=blastn&PAGE_TYPE=BlastSearch&LINK_LOC=blasthome). For genome sequencing by Eurofins Genomics, a NEBNext Ultra II DNA preparation kit was used, and Illumina NovaSeq 6000 S2 paired-end genomic sequencing was performed with a read length of 2 × 151 bp, resulting in 10,125,972 reads with a Phred score of ≥28 and a total of 1,518,897,000 sequenced bases. Additional quality control was performed to remove any remaining adapter sequences using the Trim Reads tool in the CLC Genomics Workbench v. 20.1. Assembly was performed using the CLC *de novo* assembly tool, resulting in a total sequence length of 5,736,602 bp and a total gapped length of 5,736,406 bp, distributed in 38 contigs, with an *N*_50_ value of 267,803 bp, coverage of 265×, and GC content of 59.44%. Unless otherwise noted, default parameters were used for all software. Annotation of the draft genome was done using the NCBI Prokaryotic Genome Annotation Pipeline (PGAP) v. 6.1 (https://www.ncbi.nlm.nih.gov/genome/annotation_prok). The genome completeness was estimated at 100% using CheckM v. 1.0.18 (6). A phylogenomic analysis revealed clustering within the genus *Agrobacterium* with pairwise average nucleotide identity (ANI) and digital DNA-DNA hybridization (dDDH) values of 97.97% and 83.8%, respectively, against the type strain *A. radiobacter* NCPPB 3001 using the ANI calculator (http://enve-omics.ce.gatech.edu) (7) and the Genome-to-Genome Distance Calculator v. 3.0 (https://ggdc.dsmz.de/ggdc.php#) (8, 9) with default settings. A BLAST Ring Image Generator (BRIG) v. 0.95 (10) comparison with *A. radiobacter* K84 was performed, revealing significant homology with both chromosomes of strain K84 (Fig. 1). A complete set of virulence genes guiding the transfer of transfer DNA (T-DNA) from bacteria to plant cells were identified, as well as the plasmid Ti partitioning genes, *repA* and *repB*, and the plasmid Ti replication initiator gene, *repC.* The reported data will be useful for future understanding of the genetic diversity and virulence potential of *A*. *radiobacter* in Central Asia.

### Data availability.

The partial 16S rRNA gene and whole-genome shotgun sequences of *A. radiobacter* strain MD22b have been deposited in DDBJ/ENA/GenBank under accession numbers OP364099 and JANDHV000000000, respectively. The associated BioProject, SRA, and BioSample accession numbers are PRJNA856860, SRR21562299, and SAMN29594852, respectively.
